# Anionic and zwitterionic moieties as widespread glycan modifications in non-vertebrates

**DOI:** 10.1007/s10719-019-09874-2

**Published:** 2019-07-05

**Authors:** Katharina Paschinger, Iain B. H. Wilson

**Affiliations:** Department für Chemie, Universität für Bodenkultur, 1190 Wien, Austria

**Keywords:** Glycomics, Glycans, Glucuronic acid, Phosphorylcholine, Phosphoethanolamine, Sulphate, Nematode, Insect, Mollusc

## Abstract

Glycan structures in non-vertebrates are highly variable; it can be assumed that this is a product of evolution and speciation, not that it is just a random event. However, in animals and protists, there is a relatively limited repertoire of around ten monosaccharide building blocks, most of which are neutral in terms of charge. While two monosaccharide types in eukaryotes (hexuronic and sialic acids) are anionic, there are a number of organic or inorganic modifications of glycans such as sulphate, pyruvate, phosphate, phosphorylcholine, phosphoethanolamine and aminoethylphosphonate that also confer a ‘charged’ nature (either anionic or zwitterionic) to glycoconjugate structures. These alter the physicochemical properties of the glycans to which they are attached, change their ionisation when analysing them by mass spectrometry and result in different interactions with protein receptors. Here, we focus on N-glycans carrying anionic and zwitterionic modifications in protists and invertebrates, but make some reference to O-glycans, glycolipids and glycosaminoglycans which also contain such moieties. The conclusion is that ‘charged’ glycoconjugates are a widespread, but easily overlooked, feature of ‘lower’ organisms.

## Introduction

Glycans modify a range of proteins and lipids, thereby altering their properties and their interactions. All cells have a glycan coat and so the exact nature of glycan structures can determine a wide range of cell-cell, gamete-gamete, host-pathogen, vector-pathogen and host-symbiont interactions; it can be assumed that the glycomic repertoire of a species is formed by evolutionary processes and can define speciation [1]. The basic building blocks of glycans are monosaccharides with hexoses, deoxyhexoses, pentoses and *N-*acetylhexosamines being very common regardless of whether the glycoconjugate is of animal, plant, protist, fungal or bacterial origin, although the monosaccharide repertoires of bacteria and plants are the most diverse. In addition to these ‘uncharged’ sugars, which can be modified also by moieties such as methyl groups, there are ‘charged’ saccharide and non-saccharide elements. Thereby, hexuronic and sialic acids on glycoconjugates are derived from corresponding uridine diphosphate or cytidine monophosphate donors for the relevant glycosyltransferases; in the case of sulphates, phosphate esters and phosphonates, other activated known or unknown donors are required by the enzymes that perform ‘post-glycosylational modifications’ of glycans.

While sialic and hexuronic acids are well-known in vertebrates as components of glycosaminoglycans, glycoproteins and glycolipids [2–4], sulphates are widespread on glycans of many species [5, 6]. Phosphates are known in the context of the mannose-6-phosphate marker for trafficking of lysosomal enzymes [7], but phosphodiesters are less common: exceptions being that phosphoethanolamine is a component of glycosylphosphatidylinositol anchors [8] and ribitol phosphodiesters are part of the modification of dystroglycan [9]. This contrasts with the invertebrates: sialic acids are generally rare, but phosphodiesters and phosphonates have been found on glycoconjugates in a number of species. Hexuronic acids are certainly found in invertebrates, but are less familiar or absent from fungi and protists. In plants, charged modifications (especially hexuronic acids) are on polysaccharides [10, 11], but not on N-glycans.

As N-glycans are the most commonly analysed glycoconjugates and most specific structural information in non-vertebrates is on this class of structure, we will concentrate on these. We also mention the occurrence of anionic and zwitterionic modifications in O-glycan, glycolipid and glycosaminoglycan structures, but exclude glucuronylated and sulphated small metabolites from our discussion. It is probably impossible to be exhaustive in citing all relevant literature and there are other recent reviews which also address neutral glycans in various invertebrates [12–14]. We hope readers will appreciate the overall conclusion that hexuronic and sialic acid, sulphate and phospho-based moieties in non-vertebrates can no longer be overlooked, but indeed vastly increase the range of possible glycan structures in a large number of species.

## Hexuronic acids

Glucuronic acid is probably the most common hexuronic acid; its activated form (UDP-GlcA) derives from the action of a dehydrogenase on UDP-Glc [15]. In turn, UDP-GlcA can be decarboxylated into UDP-Xyl; thus, any organism capable of making and transferring xylose can also theoretically have glucuronylated glycoconjugates. Indeed, glucuronic acid is known from a variety of glycoconjugates from different species. In terms of mass spectrometric analyses, glucuronic acid has the same mass increment (*Δm/z* 176 Da) as a methylated hexose. However, hexuronic acids, but not methylhexoses, enable ionisation in both positive and negative MALDI-TOF MS modes; also, aiding definition of these modifications are glucuronidases, which can be used in the analysis of glucuronylated glycans, as well as the enrichment of such structures in the ‘acidic’ fraction upon graphitised carbon-based solid phase extraction [16]. Other known hexuronic acids, include galacturonic acid and iduronic acid, which are respectively components of plant cell wall polysaccharides and animal glycosaminoglycans [17, 18].

**Fig. 1:**
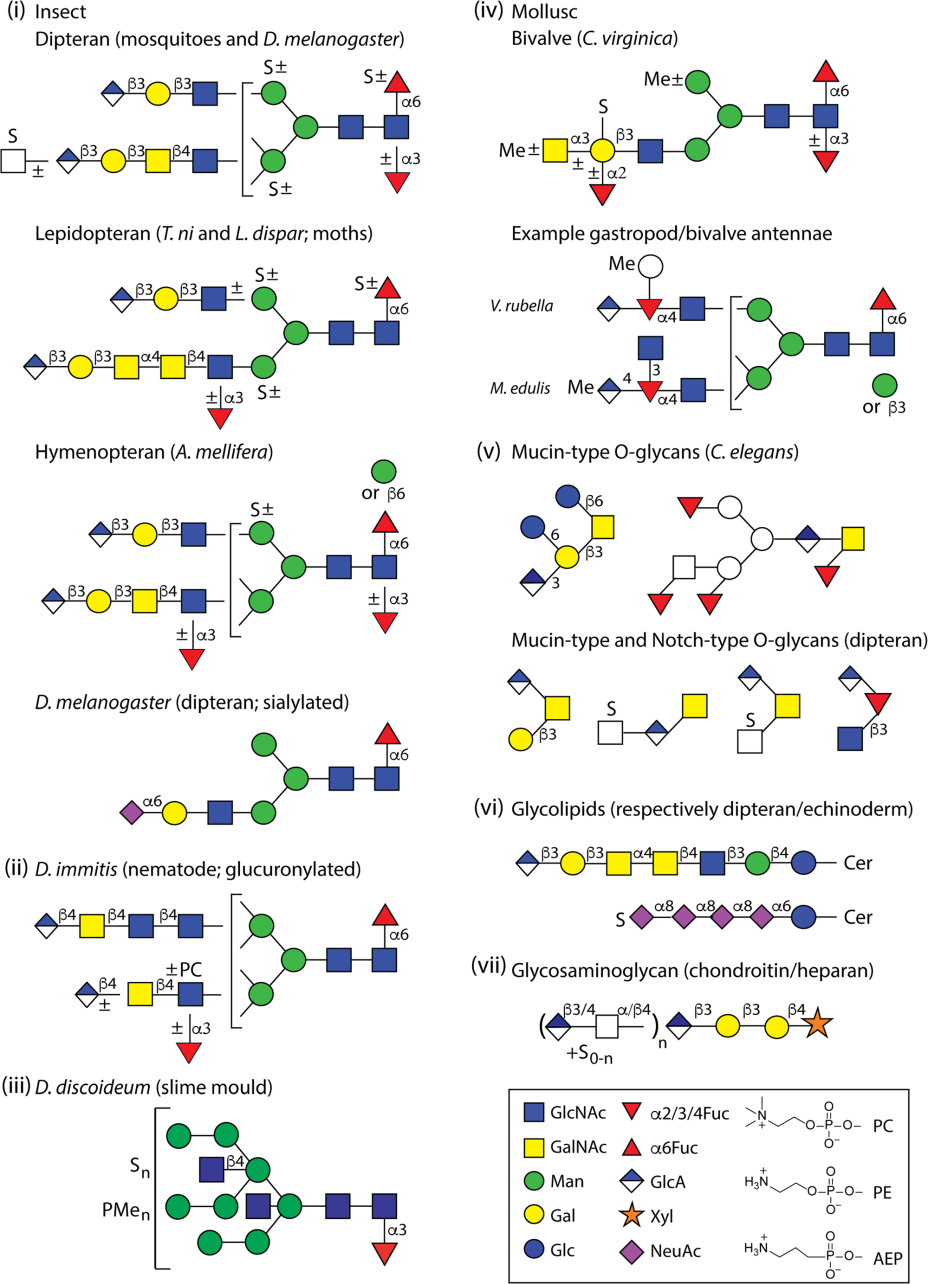
**Example glucuronylated, sialylated and sulphated glycans.** Various features of N-glycans with anionic moieties are shown to highlight the variations from (i) dipteran (*Aedes aegyptii*, *Anopheles gambiae* and *Drosophila melanogaster*), lepidopteran (*Trichoplusia ni* and *Lymantria dispar*) and hymenopteran (*Apis mellifera*) insect species, (ii) a nematode (specifically *Dirofilaria*), (iii) the cellular slime mould *Dictyostelium discoideum* and (iv) *Crassostrea virginica* (oyster), *Volvarina rubella* (marine snail; upper depicted arm) and *Mytilus edulis* (blue mussel; lower depicted arm). Also shown are (v) example ‘mucin-type’ and ‘Notch-type’ O-glycans from nematodes and insects, (vi) glycolipids from *Drosophila melanogaster* (dipteran; glucuronylated) and *Hemicentrotus pulcherrimus* (echinoderm; sialylated) and (vii) the glycosaminoglycans (the latter being common to all animals). The glycans are depicted according to the Symbolic Nomenclature for Glycans (see box); MeAEP, methylaminoethylphosphonate; P, phosphate; PC, phosphorylcholine; PE, phosphoethanolamine; PMe, methylphosphate; Pyr, pyruvate, S, sulphate; white circles or boxes indicate undefined hexoses or *N-*acetylhexosamines. Linkages are defined for proven antennal motifs, while basic trimannosylchitobiosyl cores are assumed for all N-glycans

We and others have found glucuronic acid residues on N-glycans from molluscs, insects and even a nematode (Fig. 1). In the case of molluscs (specifically one gastropod, *Volvarina rubella*, and one bivalve, *Mytilus edulis*), glucuronic acid modifies antennal fucose residues [19, 20]; thus far in insects, whether mosquitoes, the fruit fly, the honeybee or moth species, glucuronic acid was found attached to galactose [21–24] to form a non-sulphated form of the so-called HNK-1 epitope, while in one filarial nematode (*Dirofilaria immitis*) *N-*acetylgalactosamine residues are glucuronylated [25]. Thereby, it is interesting that *D. immitis* is transmitted via mosquitoes, but it is unknown whether the ‘common’ N-glycan modification with glucuronic acid is relevant to the parasite’s lifecycle. Furthermore, glucuronic acid is the anionic component of acidic glycolipids from flies [26], as part of GlcAβ1,3Galβ1,3GalNAc motifs similar to those found on N-glycan antennae from various insects, whereas GlcA-diacylglycerol is a lipid known from *Aspergillus fumigatus* [27]. Another yeast pathogen, *Cryptococcus neoformans*, expresses GlcA side chains linked to galactose residues of a glucuronoxylomannogalactan capsular polysaccharide [28].

O-glycans from insects can also be modified with glucuronic acid [22, 29, 30], whether these be of the ‘mucin-type’ (*e.g.*, GlcAβ1,3Galβ1,3GalNAc, Galβ1,3[GlcAβ1,4]GalNAc or ones carrying Hex_1_HexNAc_1_HexA_1_ repeats; the latter found in the lepidopteran HighFive cell line and in mosquitoes) or the ‘Notch-type’ (GlcNAcβ1,3[GlcAβ1,4]Fuc; see Fig. 1); recently, it was shown that O-glycan glucuronylation is important for neuromuscular junction formation in *Drosophila* [31]. Glucuronic acid is also present on O-glycans from *Caenorhabditis elegans* [32], while a circulating antigen from the trematode *Schistosoma mansoni* is a βGalNAc-based threonine-linked polysaccharide with β1,3GlcA side chains [33]. Monoglucuronylated O-glycans with reducing terminal mannose have been described from the fungus *Trichoderma reesii* [34].

Chondroitin and heparan chains of proteoglycans with varying degrees of sulphation can also be considered as O-glycans, as these primarily consist of HexA_1_HexNAc_1_ glycosaminoglycan repeats attached via a GlcAβ1,3Galβ1,3Galβ1,4Xyl tetrasaccharide linker (see Fig. 1) in probably all animals, including cnidarian, annelid, nematode and insect species [35–38]. The structurally-related dermatan sulphate containing iduronic acid is also found in echinoderms [39], while hyaluronic acid occurring as (GlcAβ3GlcNAcβ4)_n_ repeats is apparently absent from invertebrates [40]. In terms of genetic model organisms, glycosaminoglycans from *Caenorhabditis* and *Drosophila* have a number of roles in development as shown by studies on various mutants lacking key enzymes in chondroitin and heparan biosynthesis [41, 42]. Probably the best characterised invertebrate β1,3-glucuronyltransferases are three enzymes from *Drosophila* with potential to act in N-, O-, and lipid glycan or glycosaminoglycan biosynthesis [43].

## Sialic acids

In most invertebrates, it can be safely concluded that sialic acids are absent, as neither the relevant monosaccharides have been detected by modern methods nor the genetic capacity to make or transfer them is present. Insects constitute an exception as these do have sialyltransferase genes; however, only in Drosophila is there proper evidence as to it being attached to glycans [44]. On the other hand, as discussed below, primitive deuterostomes such as echinoderms have multiple sialyltransferase genes and a number of studies have proven the occurrence of sialylated glycans. However, the presence of plant genes with homology to sialyltransferases [45] also throws up questions as to the real enzymatic and biological role of such proteins in organisms for which the occurrence of sialic acid on glycans is not proven; indeed, more recently it has been proposed that these plant sialyltransferase-like enzymes may transfer either 2-keto-3-deoxy-D-lyxo-heptulosaric acid (DHA) or 2-keto-3-deoxy-D-manno-octulosonic acid (KDO) to rhamnogalacturonan-II [46]. In the case of trypanosomatids, trans-sialylation results in parasites coating their mucins with host-derived sialic acid [47]. Various reports claiming that sialic acid is present in other invertebrate organisms, however, must be taken with caution, as mere HPLC peaks or lectin blotting data are not conclusive proofs and can be due to, *e.g.*, lectin cross-reactivity or the presence of contaminants; only mass spectrometric proof of the structure, in the absence of deuterostome components in media or food, is an acceptable demonstration of sialylation.

Sialylation in insects has long been controversial. One early report suggested the presence of polysialic acid in *Drosophila* [48], but the only sialylated glycans found by mass spectrometry contain a single residue of *N-*acetylneuraminic acid attached to galactose on N-glycans [44, 49] (see example in Fig. 1). The single *Drosophila* sialyltransferase has been functionally and biochemically characterised [50, 51]. In other insect species, sialyltransferase genes have been identified and parts of the CMP-sialic acid biosynthesis pathway revealed [52, 53]; however, only upon glycoengineering can it be safely concluded that Lepidoptera will express sialic acid on recombinant glycoproteins [54].

In the deuterostome lineage, even in organisms evolutionarily more primitive than the vertebrates, there is quite definitely an expansion of the sialyltransferase gene family. For instance, multiple sialyltransferases are encoded by the genome of a cephalochordate (*Branchiostoma belcheri*); both *N*-acetylneuraminic acid (Neu5Ac) or *N*-glycolylneuraminic acid (Neu5Gc) were found to be expressed in a tissue-specific manner in this organism, while methylated 3-deoxy-D-glycero-D-galacto-2-nonulosonic acid (KDN; a non-acylated sialic acid) was found on ovary O-glycans [55]. In echinoderms, a number of α2,3-, α2,6- and α2,8-sialyltransferase homologues, yet to be enzymatically characterised, occur in the sea urchin *Strongylocentrotus purpuratus* [56–58]; NeuAc and NeuGc are found in a number of echinoderm species, also in *O*-acetylated and *O*-sulphated forms. In starfish and sea cucumbers, a large percentage of these sialic acids are additionally methylated at O-8, whereas in other echinoderms this position can be sulphated [59, 60]. Relevant metabolic enzymes for the generation of methylated NeuGc are the CMP-NeuAc hydroxylase, as also found in non-human mammals, and an 8-*O*-methyltransferase [61, 62].

Specific examples of defined sialylated structures in echinoderms include a mucin-type O-glycan from the sperm flagella of the sea urchin (*Hemicentrotus pulcherrimus*) which contains terminal sulphated O-8 α2,9-linked polyNeu5Ac linked to the protein backbone via GalNAc [63, 64], a motif also present in two other sea urchins (*Strongylocentrotus purpuratus* and *Str. franciscanus*). On other sea urchin sperm and egg O-linked glycoproteins, α2,8-sialic acid and α2,5-sialic acid were found [65, 66]. Gangliosides (i.e., lactosyl- or glucosylceramides carrying one or more NeuAc or NeuGc residues, reportedly also in fucosylated, methylated or sulphated forms; see example in Fig. 1) represent another class of sialylated glycoconjugates studied in species of various echinoderm classes [67–71], such as sea cucumbers (*Stichopus chloronotus*, *Sti. japonicus* and *Holothuria leucospilota*), starfish (*Linckia laevigata, Asterias amurensis, Luidia maculata, Asterina pectinifera* and *Acanthaster planci*), sea urchins (*Str. intermedius* and *H. pulcherrimus*), a feather star (*Comanthus japonica*) and a brittle star (*Ophiocoma scolopendrina*).

## Pyruvylation

The presence of pyruvate on N-glycans is known from yeast species, specifically 4,6-ketal-linked to outer chain galactose residues [72], whereby it should also be noted that some bacterial and seaweed polysaccharides are also pyruvylated. The *pvg1* gene, a pyruvyltransferase, is one of five necessary for pyruvylation in the fission yeast, whereby the *pvg3* gene encodes the galactosyltransferase required for transfer of the underlying β1,3-linked galactose residue [73, 74]. Pyruvate was also found on glycolipids of the sea hare, which is a mollusc [75] and on the glyconectin polysaccharides isolated from some *Porifera* sponges [76].

## Sulphate

Probably sulphation is most familiar from glycosaminoglycans (*e.g.*, heparan, chondroitin and keratan sulphates), but some of these are either absent or undersulphated in many invertebrates [77]; on the other hand, some marine organisms have highly sulphated glycosaminoglycans, sometimes also with fucosyl modifications [78]. Indeed, an increased degree of sulphation on various glycoconjugates seems to occur in the context of marine or saline environments [79, 80]. Especially glycosaminoglycans from sharks, hagfish, king crabs, squid, molluscs, sea cucumbers and sea squirts are characterized by oversulphated complex structures which carry more sulphated residues per disaccharide unit than the terrestrial vertebrates on their chondroitin and dermatan sulphates [77], which contain only one sulphate group per disaccharide unit (one, two or three sulphates being possible, of course, on the disaccharide units of vertebrate heparan sulphate). Sulphated fucans and galactans from echinoderms, distinct from glycosaminoglycans, may have roles in fertilisation [81, 82]. Also, although sulphated polysaccharides have not been described in vascular plants (angiosperms), sulphated galactans and fuc(oid)ans have been isolated from marine sea grass species and algae [79, 80], while sulphoquinovosyldiacylglycerol is an important anionic glycolipid component of plant chloroplasts [83]. Sulphated O-glycans with mannose at the reducing terminus have been described from a coral [84].

In terms of N-glycans, sulphation of α-linked mannose and/or core α1,6-fucose is known from arthropods (specifically a lobster as well as insects; see examples in Fig. 1) and from *Dictyostelium* [21, 85–88], while sulphation of galactose is a feature of the Eastern oyster [89]. Based on studies with radioactive SO_4_ labelling and PNGase F treatment, the occurrence of sulphated N-linked oligosaccharide chains on glycoproteins involved in sea urchin embryonic skeleton formation was postulated [90]; these glycan chains, recognised by the 1223 antibody, apparently bind calcium [91], but their exact structures were not defined. Sulphated ‘mucin-type’ O-glycans are known from a marine snail as well as from dipteran species, whereby the major structures are based on the core 1 Galβ1,3GalNAc motif [20, 21]. More unusual are the sulphated O-glycans with Hex-HexNAc-HexA repeats found in mosquito larvae and a recombinant protein expressed in HighFive cells [21, 30]. As mentioned above, 8-*O*-sulphated sialic acid has been found on sea urchin O-glycans and glycolipids [63, 70]. Generally, the sulphate on glycans originates from 3′-phosphoadenosine-5′-phosphosulfate as the activated donor; however, other than sulphotransferases involved in glycosaminoglycan synthesis [92–96], probably no other invertebrate glycan-modifying sulphotransferases have been either biochemically or biologically characterised.

## Phosphate and methylphosphate

**Fig. 2:**
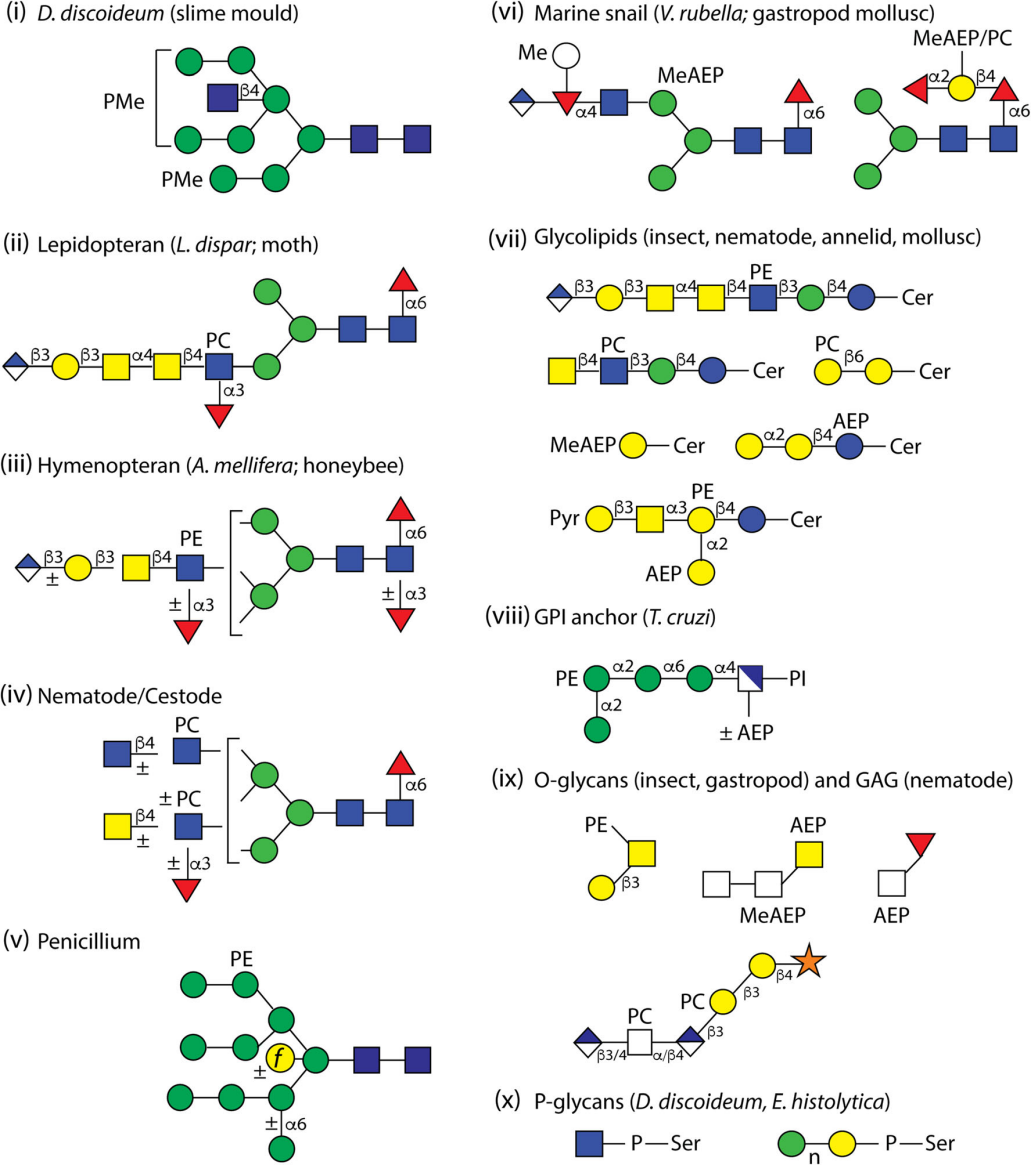
Example zwitterionic and phosphodiester structures. The depicted structures are (i) a methylphosphorylated N-glycan structure from *Dictyostelium discoideum*, (ii) an N-glycan from *Lymantria dispar* modified with glucuronic acid and phosphorylcholine, (iii) a schematic honeybee N-glycan, (iv) a schematic glycan from either nematode or cestode species, (v) a phosphoethanolamine-modified N-glycan from *Penicillium* spp. optionally with an ‘outer chain’ mannose and a bisecting galactofuranose, (vi) two N-glycans with methylaminoethylphosphonate or phosphorylcholine from *Volvarina rubella,* (vii) glycolipids from insects (example from *Drosophila*), nematodes and annelids (with phosphorylcholine) and molluscs (with aminoethylphosphonate; also observed with pyruvate and phosphoethanolamine modifications), (viii) a glycosylphosphatidylinositol (GPI) anchor from the *Trypanosoma cruzi* NETNES protein, (ix) a mucin-type O-glycan from wasp, a mucin-type and a Notch-type from *Volvarina rubella*, a glycosaminoglycan-like glycan from *Oesophagostomum dentatum* and (x) phospho-linked sugars from either *Dictyostelium discoideum* or *Entamoeba histolytica.* AEP, aminoethylphosphonate; MeAEP, methylaminoethylphosphonate; P, phosphate; PC, phosphorylcholine; PE, phosphoethanolamine; PMe, methylphosphate; Pyr, pyruvate

In mammals, mannose-6-phosphate is well known for its role in trafficking of lysosomal enzymes; defects in the two-step phosphorylation process (first transfer of GlcNAc-1-phosphate in 6-linkage to mannose, then removal of the GlcNAc to reveal mannose-6-phosphate) result in mislocalisation of lysosomal hydrolases and so to forms of lysosomal storage diseases [97]. Three decades ago, GlcNAc-1-phosphotransferase activities were found in amoebae [98]; more recently the relevant *gpt1* gene encoding this enzyme from *Dictyostelium* has been identified [99]. The final glycan structures in *Dictyostelium discoideum*wild-type and mannosyltransferase mutant strains contained rather methylated phosphate attached to mannose [88, 100] (see Fig. 2). In a glucosidase II amoebal mutant, however, there was also a significant amount of unmodified and GlcNAc-modified phosphate [101, 102], which is indicative that the biosynthetic route to the methylphosphorylated structures is affected by other aspects of glycan processing. A phosphorylated Man_4_GlcNAc_2_ was also found in one strain of *Trichomonas vaginalis*, a human parasite [103]; it is unclear whether this is due to loss of an ethanolamine from a phosphoethanolamine-modified structure otherwise found in this species. Another form of mannosyl phosphorylation is represented by the mannose-phosphodiesters found in hypermannosylated structures from *Saccharomyces cerevisiae* and originates by transfer from GDP-Man rather than by a kinase reaction [104, 105]; other phosphodiester-type glycans include the phosphoglycan on the gp72 of *Trypanosoma cruzi*, the polyhexose chains on the lipoproteophosphoglycan of *Entamoeba histolytica* and the GlcNAc-P-Ser motif on proteins from *Dictyostelium discoideum* [106–108] (for the latter two examples, see Fig. 2)*.* In terms of analyses, phosphorylated glycans are well observed in positive and negative modes of mass spectrometry and the phosphates can be removed by hydrofluoric acid or (if not methylated) by phosphatases [16].

## Phosphorylcholine and phosphoethanolamine

Other than phosphate, zwitterionic phosphodiesters modify glycoconjugates from a range of prokaryotic and eukaryotic organisms. Phosphoethanolamine (PE; also known as aminoethylphosphate or ethanolamine phosphate; *Δm/z* 123 Da) and its tri-*N*-methylated form, phosphorylcholine (PC; *Δm/z* 165 Da), have been described on N-glycans, O-glycans, glycolipids and glycosaminoglycans from protists, fungi, insects, annelids and nematodes as well as on lipopolysaccharides from various bacteria. Phosphoethanolamine is also a component of eukaryotic glycosylphosphatidylinositol (GPI) anchors [109].

In nematodes and in one cestode it appears that GlcNAc or GalNAc residues of N-glycans and glycolipids can be modified with phosphorylcholine (see examples in Fig. 2), as shown by studies on *C. elegans*, *Ascaris suum*, *Trichuris suis*, *Haemonchus contortus*, *Oesophagostomum dentatum*, *Pristionchus pacificus, Trichinella spiralis*, various filarial worms (including *Dirofilaria immitis*) and *Echinococcus granulosus* [25, 110–119]; the underlying structures of the phosphorylcholine-modified N-glycans from nematodes vary, but multiple residues (in the context of a single GlcNAc, chito-oligomer or LacdiNAc-type motifs) on two, three or four antennae are possible. Phosphorylcholine is also found on annelid and nematode glycolipids [120–123], on glycosaminoglycan-like structures from at least one nematode [124] and on O-glycans with the composition HexNAc_2-3_HexA_1_PC_1_ released from a recombinant protein expressed in lepidopteran (Sf9) cells [30]. In fungi, phosphorylcholine is found to substitute either galactofuranose on glycosylinositol-phosphoceramide glycolipids [125] or mannose in a peptidophosphogalactomannan [126].

Phosphorylcholine was found on core ‘GalFuc’ moieties of N-glycans of the gastropod *Volvarina rubella* [20] as well as on the antennae of a not insignificant modification of N-glycans from Lepidoptera, including cell lines used in biotechnology such as *Trichoplusia ni* HighFive [24]. In other insects, it is rather phosphoethanolamine that is found on N-glycans of the honeybee [23] and the O-glycans of wasps [127]; on the other hand, N-glycans from Diptera were not yet found to contain zwitterionic modifications, even though phosphoethanolamine linked to GlcNAc is a component of glycolipids from dipteran insects such as *Drosophila* and *Calliphora* [128, 129] (see example in Fig. 2) or on O-glycans from mosquito larvae with the composition HexNAc_1-2_Hex_1_HexA_2_PE_1_ [21]. Phosphoethanolamine is also present on mannose residues of N-glycans from *Penicillium* species and *Trichomonas vaginalis* [103, 130] and has been reported as a modification of glycolipids from the bivalve, *Corbicula sandai* [131].

At the analytical level, phosphoethanolamine yields signals in both positive and negative modes of MALDI-TOF MS, while the phosphorylcholine is only (and excellently) detectable in positive mode [16]; however, both are sensitive to hydrofluoric acid hydrolysis. The enzymatic basis for addition of these moieties is far from resolved, but conceivably CDP-choline/ CDP-ethanolamine or phosphatidylcholine/ phosphatidylethanolamine could be the donor substrates for PC/PE-transferases [132–134]. Both phosphorylcholine and phosphoethanolamine are ligands for pentraxins (*e.g.*, human C-reactive protein and serum amyloid P), which are components of the innate immune system [135]; at least phosphorylcholine modifications on glycolipids and glycoproteins are associated with immunomodulatory activity, as especially shown for the *Acanthocheilonema viteae*ES-62 protein, which is an excretory-secretory protein of a rodent nematode parasite, whereby the Toll-like receptor 4 (TLR4) may be involved in the relevant signalling pathway [136].

## Aminoethylphosphonate

Phosphonates differ from phosphates in that there is a carbon-phosphorus bond without any intermediate oxygen atom. In eukaryotes, the first demonstrations of the 2-aminoethylphosphonate and *N-*methyl-2-aminoethylphosphonate modifications of glycans (*Δm/z* 107 and 121 Da) came from studies on glycolipids of gastropods, such as the sea hare *Aplysia kurodai* [137–142]; as shown in Fig. 2, one such glycolipid is reported to also contain pyruvate and phosphoethanolamine residues [75]. In other studies, a GPI anchor from *Trypanosoma cruzi* (the causative agent of Chagas’ disease), N-glycans from locust and O-glycans from jellyfish were found to be modified with aminoethylphosphonate [143–145]. Most recently, methylaminoethylphosphonate and aminoethylphosphonate were detected on N-glycans as well as O-fucose-based structures from a marine snail [20], while there is also evidence that oligomannosidic glycans from *Euglena gracilis*, a free-living protist, carry aminoethylphosphonate [145].

Predominantly, it seems that either mannose or GlcNAc residues can be modified by these phosphonates; like phosphorylcholine and phosphoethanolamine, these can be cleaved by hydrofluoric acid. Aminoethylphosphonates were detected in both positive and negative ion modes of FAB- and MALDI-TOF-MS. Neither the recognition of aminoethylphosphonate by pentraxins nor the routes for its transfer to glycan substrates is known.

## Mass alone is not enough

**Fig. 3:**
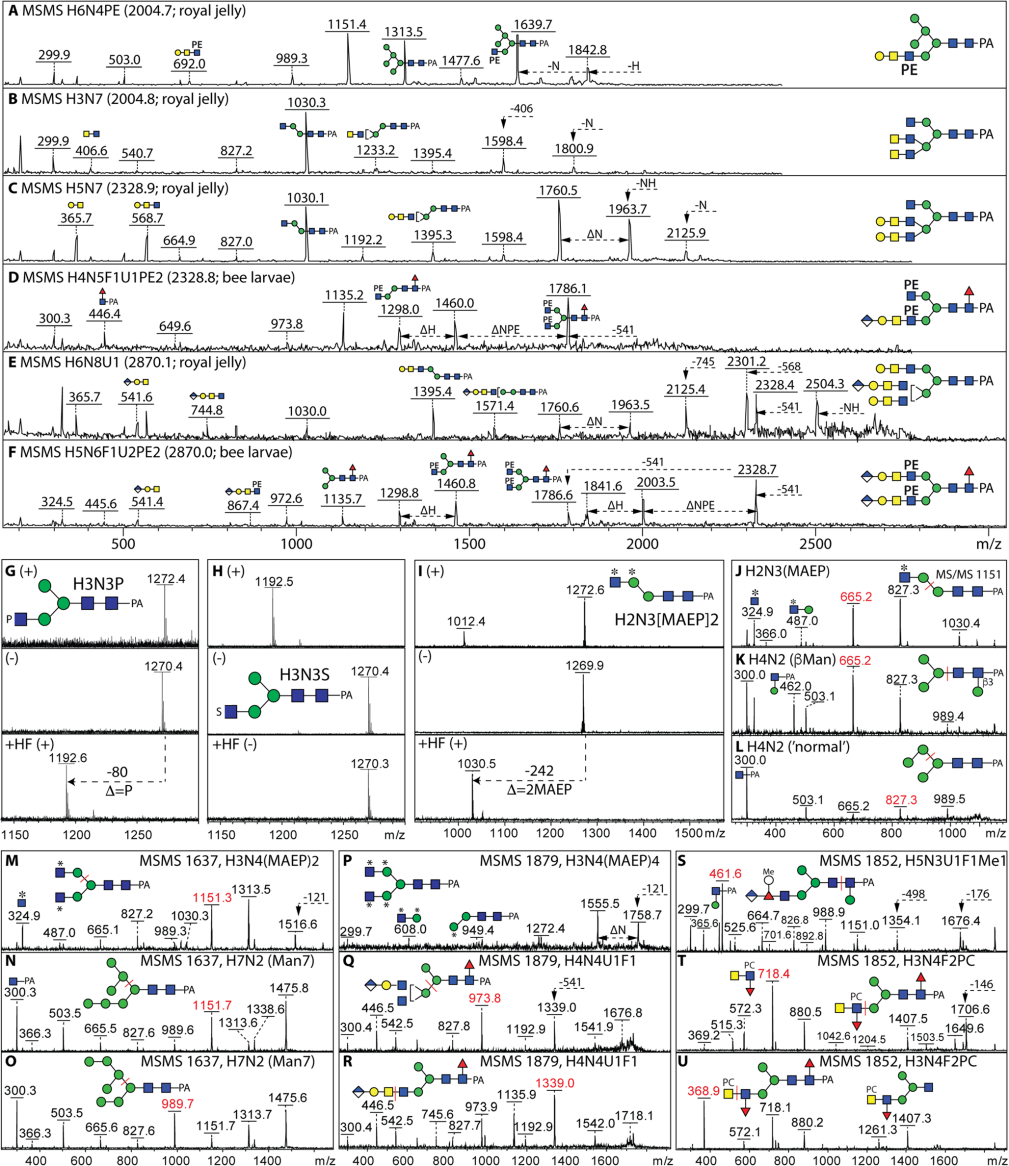
**Mass spectrometry of isobaric/isomeric structures. a-f** Example positive mode MS/MS of N-glycans from *Apis mellifera* royal jelly or larvae which isobarically differ depending on the presence of phosphoethanolamine, LacdiNAc or glucuronylated motifs. **g-i** Example positive (+) or negative (−) mode MS of isobaric glycans from an echinoderm or *Volvarina* with masses of 1271 Da carrying either phosphate, sulphate (note in-source loss in positive mode) or methylaminoethylphosphonate moieties and are distinguishable due to their ionisation in positive mode or sensitivity to hydrofluoric acid (HF). **j-l** Positive mode MS/MS of isobaric/isomeric variations of 1150 Da (*m/z* 1151) from *Volvarina rubella* either methylaminoethylphosphonate-modified, core β-mannosylated or ‘normal’ paucimannosidic. **m-u** Positive mode MS/MS of variations of glycans of *m/z* 1637, 1879 or 1852 which are either methylaminoethylphosphonate-modified (*Volvarina*), glucuronylated (*Apis*), phosphorylcholine-modified (*Trichoplusia* or *Dirofilaria*) or standard oligomannosidic structures. Annotated are the key fragments (symbolic nomenclature), losses (with arrows) or cleavages (red bars and *m/z* values); all glycans were reductively aminated with 2-aminopyridine (PA) which yields typical reducing terminal Y fragments of *m/z* 300, 446 or 462 (GlcNAc_1_Fuc_0–1_Man_0–1_-PA). Abbreviated compositions of the form H_x_N_y_F_0–1_ U_0–1_ correspond to Hex_x_HexNAc_y_Fuc_0–1_HexA_0–1_; MEAP (or *), methylaminoethylphosphonate; PC, phosphorylcholine; PE, phosphoethanolamine; S, sulphate

Perhaps part of the reason for the historically relatively low numbers of proven anionic and zwitterionic glycans in non-vertebrates is that they were not expected. Also, in screens based on positive mode mass spectrometry alone without fragmentation, many such structures would not be noticed as they are isobaric (i.e., same mass) as compared to more well-known structures; most commonly, a typical mass spectrometer without a very high resolution will not distinguish a phosphate from a sulphate. Combined with enzymatic or chemical treatments of glycans, careful assessment of MS/MS fragments is indeed required, as perhaps only a couple of fragments are showing the structural difference. For instance, MS/MS is necessary to show that a neutral complex N-glycan is not actually carrying phosphoethanolamine (see examples for *m/z* 2004, 2328 and 2870; Fig. 3 a-f) or a glycan with the same mass as a oligomannosidic glycan is actually one modified with methylaminoethylphosphonate on antennal GlcNAc (see the examples for *m/z* 1151 and 1637; Fig. 3 j-o); other examples offered by the marine snail *Volvarina rubella* include (i) a glycan with four methylaminoethylphosphonate residues isobaric (*m/z* 1879; Fig. 3 p) with glucuronylated structures from insects (Fig. 3 q and r) or (ii) a glycan with a bisubstituted fucose residue with the same mass (*m/z* 1852; Fig. 3 s) as structures from insects or nematodes carrying phosphorylcholine (Fig. 3t and u). As we have previously reviewed in this journal [16], not only separation of classes and isomers of glycans is required, the different ionisation and fragmentation in positive and negative modes of mass spectrometry is useful in showing whether a glycan is indeed that ‘predicted’ from the mass, while hydrofluoric acid hydrolysis is valuable as it will remove phosphate esters (Fig. 3 g-i), fucose (especially in α1,3-linkage) and galactofuranose, but not sulphate or other hexoses.

## Conclusion

In this brief overview of anionic and zwitterionic glycans from various lower eukaryotes, it is obvious that the glycosylation in protists and invertebrates is highly variable. Of course, our knowledge is only based on those organisms or phyla which have been investigated; very many phyla (and naturally huge numbers of species) have never been studied (or adequately so) as to their glycomes. Mere mass spectrometric profiling, without doing deep analyses based on fractionated glycomes, will also not show the true extent of the glycostructural possibilities. As many anionic or zwitterionic glycans with potential biological activity occur in low amounts, they are often overseen if, *e.g.*, the whole N-glycome is ‘shot’ from a single spot on a MALDI plate; certainly the isomeric structures will not be easily distinguished. However, as technologies and methodologies evolve, there is potential for even more variants and/or commonalities between species.
